# Postgraduate medical trainees at a Ugandan university perceive their clinical learning environment positively but differentially despite challenging circumstances: a cross-sectional study

**DOI:** 10.1186/s12909-023-04933-7

**Published:** 2023-12-15

**Authors:** Paul E. Alele, Joshua Kiptoo, Kathleen Hill-Besinque

**Affiliations:** 1 Department of Pharmacology and Therapeutics, Mbarara University of Science and Technology, Mbarara, Uganda; 2 Department of Pharmacy, Mbarara University of Science and Technology, Mbarara, Uganda; 3 Department of Pharmacy Practice, School of Pharmacy, Chapman University, California, USA

**Keywords:** Postgraduate, Medical, Trainees, Learning, Environment, Career, Engagement, Uganda

## Abstract

**Purpose:**

The clinical learning environment is an essential component in health professions’ education. Data are scant on how postgraduate trainees in sub-Saharan Africa perceive their medical school learning environments, and how those perceptions contribute to their engagement during training, their emotional wellbeing, and career aspirations. This study examined perceptions of postgraduate medical trainees (residents) in a resource-limited setting, regarding their learning environment and explored perceptual contributions to their career engagement during training. The data reported contribute to understanding how clinical learning environments can be improved in low-resource settings in Uganda and elsewhere.

**Methods:**

This study was done at the Faculty of Medicine of Mbarara University of Science and Technology in Uganda. We used a descriptive cross-sectional design involving sequential mixed methods. Quantitative data were collected using the Postgraduate Hospital Educational Environment Measure (PHEEM). Qualitative data were collected using focus group discussions.

**Results:**

Ninety of the 113 eligible residents responded (79.6%). Of these, 62 (68.9%) were males, 51 (56.7%) were third-year trainees, and the majority (40%) of the residents were aged between 30 and 34 years. Overall PHEEM scored 98.22 ± 38.09; Role Autonomy scored 34.25 ± 13.69, Teaching scored 39.7 ± 13.81, and Social Support scored 24.27 ± 10.59. Gender differences occurred in the perceptions of teaching and social support. Cronbach’s alpha coefficient was 0.94 for the overall PHEEM. Five major themes were identified from the qualitative data (trainee support, supervision environment, engagement with overall learning environment, preparation for future practice, and challenges that impede training).

**Conclusions:**

Overall, this study suggests that postgraduate trainees at the institution perceived the clinical learning environment positively amidst challenges of limited resources. Trainees’ insights provided data that propose improvements on a number of domains in the learning environment.

## Introduction

Postgraduate medical trainees’ perceptions of their learning environment (LE) relative to emotional wellbeing, engagement, and career satisfaction are important as individuals navigate through training. Learning environments are complex, comprising not just the physical and virtual spaces where learning takes place, but also the sociocultural interactions necessary for learning [1]. Research shows that medical professionals commonly experience negative states such as burnout beginning as early as during medical school training [2]. Some studies done in Western countries and in low-resource settings have shown that burnout affects between one-third to half of medical school trainees [3–6]. Burnout may lead to emotional exhaustion, depression, and alienation, significantly impairing patient care because of the reduced quality of medical care and medical errors by trainees and health professionals [3, 4].

Burnout in medical students can be mitigated by the learning environment [7, 8]. Dyrbye and colleagues, and others, have outlined strategies that can aid institutions and training sites as their educators work to improve factors related to improving the wellbeing of learners and their learning environment [9–11]. Negative LEs include inadequate role models, high student or resident workload, heavy faculty workloads, lack of feedback or poor feedback, and mistreatment during training; these can impede learning, career aspirations, and professional identity formation. Despite the importance of the LE in health professions education, there is insufficient information on how postgraduate trainees in sub-Saharan Africa perceive their medical school learning environments, and how those perceptions contribute to their engagement during training, their emotional wellbeing, and career aspirations.

Data are scant on the extent of support that postgraduate medical trainees in Uganda and other resource-limited settings receive during their academic training from their supervisors. Data are also scant on how other modifiable factors such as physical infrastructure and training frameworks may be leveraged to enhance trainees’ experience of clinical care and research. A few studies from South Africa, Ethiopia, and Kenya have explored factors associated with the learning environment, including social support [12–14]. In the study of the clinical learning environment of an internal medicine program in Ethiopia, significant challenges were identified in the learning environment as requiring immediate consideration. These included excessive trainee workload, insufficient teaching, inadequate hospital physical infrastructure, and lack of diagnostic and treatment facilities [12]. Residents in the study from Kenya at the largest national teaching and referral hospital, perceived a more positive learning environment, with the majority endorsing high social support [13].

Our specific aim in this study, therefore, was to examine how postgraduate medical trainees in a resource-limited setting perceive their learning environment and how those perceptions contribute to their engagement during training. This study will contribute to the data on the role of the learning environment in postgraduate medical trainees’ engagement, career aspirations, and wellbeing during training in Uganda and other resource-limited settings.

## Methods

### Study design and setting

We conducted a descriptive cross-sectional study using mixed methods at the Faculty of Medicine of Mbarara University of Science and Technology (MUST), a public university in Uganda, from January 12 to March 5, 2023. The Faculty of Medicine trains both undergraduate and graduate medical students. The medical curriculum for postgraduate training covers at least 3 years post-internship. Presently, postgraduate medical training is offered in all the major clinical and specialty disciplines including the Master of Medicine in Anesthesia, Community Practice/Family Medicine, Dermatology, Ear, Nose and Throat, Emergency Medicine, Internal Medicine, General Surgery, Obstetrics and Gynecology, Ophthalmology, Pediatrics and Child Health, Pathology, Plastic and Reconstructive Surgery, Psychiatry, and Radiology. A mentored research project is typically started towards the end of the second year, culminating in a dissertation which is required before graduation.

### Participants and study procedure

Potential study participants were graduate medical trainees who had completed at least 1 year of training and consented to participate in this study. A list of potential participants and their e-mails (routinely collected as part of their academic enrolment) was obtained from the different departments and specialty units through the participants’ resident representative. The survey tool and FGD interview guide were pilot-tested on four recent clinical faculty graduates who were not part of the study to ensure clarity and intended meaning were achieved. Participants for the PHEEM survey were selected by an initial e-mail invitation that was sent to all potential participants with a link to the PHEEM tool and the informed consent form, if they decided to participate. E-mail reminders were sent out to potential participants who had not completed the survey one and 2 weeks after the initial e-mail. The FGDs were done after completing the survey.

### Participant inclusion and exclusion criteria

All postgraduate medical trainees were included if they had completed at least one academic semester of training, were willing to sit for an FGD of 45 minutes to 1 hour, and consented to participating in the study. Trainees who were ill, unable to provide written consent, or were doctoral (Ph.D.) students were excluded. Doctoral students were excluded because their program of study in this setting, unlike that of the clinical postgraduates in this study, is mainly oriented toward academic research, and follows a less structured independent program since they have already attained their Master’s degree qualification.

### The quantitative data collection instrument

The PHEEM is now the most widely used and recognized tool for assessing the clinical learning environment worldwide [15]. It has been tested across a wide range of clinical specialties and has been shown to be reliable, valid, and reproducible in multiple studies worldwide [16–19]. This tool consists of 40 specific items with 3 subscales that assess role autonomy, perceptions of teaching, and perceptions of social support. Items were scored on a 5-point Likert scale (“strongly disagree” with a score of zero, to “strongly agree” with a score of 4), yielding a maximum score of 160. Three items (items 3, 38, and 39) were reverse-scored because of the negative nature of the statements, so that “strongly disagree” scored 4 and “strongly agree” scored 0. The original study by Roff and colleagues suggests interpreting the findings as: Excellent = score>120; More positive than negative, with room for improvement = 80–120; Plenty of problems = 40–80; Very poor = 0–40 [15].

### Sample size considerations

There were 113 eligible participants identified. Due to this relatively small pool of potential participants who could be included in the study, the PHEEM survey was a census of all eligible postgraduate medical trainees in the Faculty of Medicine.

### Focus group discussions

Two FGDs were conducted – the first one comprised 12 members from the departments of Psychiatry, Pediatrics, Pathology, and Surgery. The second FGD comprised 10 members from the departments of Internal Medicine and Obstetrics and Gynecology. Participants for the FGDs were selected by an email invitation that was sent to all the eligible potential participants after they completed the PHEEM survey. Those who accepted to participate were contacted by the facilitator who organized the consenting, date, time, and venue for the FGDs. The FGDs were conducted among trainees who had completed the PHEEM survey to gain a deeper understanding of their perceptions of the clinical learning environment. Data saturation was achieved by the end of the second FGD so a third one was deemed unnecessary. The FGDs were conducted at the University campus in a quiet, safe, private, well-lit and comfortable room in the Faculty of Medicine. The facilitator for the FGDs was a neutral person who at the time was not involved in teaching, tutoring, or mentoring the postgraduate medical trainees.

### Quantitative data analysis

Descriptive statistics were used to describe percentages, means and their standard deviations, and interquartile ranges of the scores in the instrument used. Internal reliability of the PHEEM tool was assessed using Cronbach’s alpha coefficient. Parametric data were analyzed using one-way analysis of variance (one-way ANOVA) while non-parametric data were analyzed using Kruskal-Wallis test with Dunn-Bonferroni correction done for further pairwise comparison to determine which pairs of categories had statistically significant differences. A *p*-value less than 0.05 was considered statistically significant. Data were analysed using GraphPad Prism version 7 and Statistical Package for the Social Sciences (SPSS), version 26 (IBM Corp, Armonk, NY, USA).

### Qualitative data analysis

Thematic content analysis was used to analyse the transcribed anonymized data from the FGDs, based on the six-step framework by Braun and Clarke [20]. Transcribed data were organized and codes developed by labelling the data into categories with related words, phrases, sentences, or sections pertaining to the objectives. To organize the data-coding a codebook was developed; the codes were checked to choose the most important codes. Categories were then generated by grouping several codes together. Study themes were created by recognizing the connections between the categories based on the study objectives, each of which had its own themes. Essential information from the FGDs was summarized by collecting important quotations regarding identified themes and objectives. Subthemes identified during this process were also added. This analytical process enabled naturally occurring themes to be identified from the data in an on-going, iterative process in parallel with data collection. Thematic code revision was done as new codes and subthemes were identified, until thematic saturation was achieved.

## Results

### The PHEEM survey

Out of 113 residents invited to participate in the survey, 90 (79.6%) responded and completed the questionnaire. Of these, 62 (68.9%) were male, 51 (56.7%) were year 3 trainees, and the majority of participants were aged between 30 and 34 years. Participants represented 12 clinical departments that train specialists for a three-year period; at the time of the study only second- and third-year postgraduate trainees met the criteria for inclusion into the study, as the first-year trainees had just commenced their first semester of training. Socio-demographic data for the participants are shown in Table 1.

**Table 1:** Socio-demographic characteristics of postgraduate trainees

Characteristic	*N* (%)	Male	Female
**Gender**			
Male	62 (68.9)		
Female	28 (31.1)		
**Age category (years)**			
25–29	19 (21.1)	10	9
30–34	40 (44.4)	26	14
35–39	23 (25.6)	20	3
40–44	5 (5.6)	4	1
45–49	3 (3.3)	2	1
**Year of training**			
Postgraduate year 2	39 (43.3)	26	13
Postgraduate year 3	51 (56.7)	36	15
**Source of funding for trainees’ education**			
Self	54 (60)	39	15
Government of Uganda Scholarship	16 (17.8)	11	5
Other	20 (22.2)	12	8
**Postgraduate specialty training**			
Anesthesiology and Critical Care	5 (5.6)	3	2
Dermatology	9 (10)	6	3
Ear, Nose, and Throat	5 (5.6)	3	2
Emergency Medicine	7 (7.8)	7	–
General Surgery	14 (15.6)	13	1
Internal Medicine	5 (5.6)	4	1
Obstetrics and Gynecology	10 (11.1)	9	1
Ophthalmology	2 (2.2)	1	1
Pathology	3 (3.3)	2	1
Pediatrics and Child Health	10 (11.1)	2	8
Psychiatry	8 (8.9)	5	3
Radiology	4 (4.4)	2	2
Undeclared	8 (8.9)	4	4

Cronbach’s alpha coefficient for the overall PHEEM tool was 0.94, for the role autonomy subscale was 0.83, for the teaching subscale was 0.93, and for the social support subscale, it was 0.74. A chi-square test of independence showed that there was no significant association between gender and age category, *X*^*2*^ (1, *N* = 90) = 6.4, *p* = .169; or gender and year of postgraduate training, *X*^*2*^ (1, *N* = 90) = 0.1586, *p* = .69.

The mean total PHEEM score was 98.22 ± 38.09, with the three sub-scales scoring 34.25 ± 13.69 for Role Autonomy, 39.7 ± 13.81 for Teaching, and 24.27 ± 10.59 for Social Support (Table 2). The items that scored the lowest were question 2: “I have a contract of employment that provides information about hours of work” (Mean, 0.85 ± 0.94), question 30: “There are adequate catering facilities when I am on call” (Mean, 1.11 ± 0.99), and question 33: “This hospital has good quality accommodation for residents, especially when on call” (Mean, 0.66 ± 0.91).

**Table 2:** Summary results for PHEEM items

Item	Male Mean ± S.D.	Female Mean ± S.D.
1. My workload in this residency is fine	2.2 ± 1.1	2.5 ± 1.1
2. I have a contract of employment that provides information about hours of work	0.77 ± 0.88	0.93 ± 1.0
3. I have to perform inappropriate tasks	2.4 ± 1.2	2.6 ± 1.1
4. There is accurate, unit specific written information available	2.0 ± 1.4	1.6 ± 0.84
5. My hours conform to the curriculum	1.8 ± 1.2	2.0 ± 1.2
6. I work according to a fixed timetable	1.9 ± 1.4	2.2 ± 1.0
7. I had an informative orientation program	2.1 ± 1.2	2.4 ± 1.2
8. There are clear clinical protocols in this rotation	2.3 ± 1.2	2.3 ± 0.93
9. The training in this rotation makes me feel ready for the next step	3.1 ± 0.79	3.1 ± 0.54
10. I have the appropriate level of responsibility in this rotation	3.1 ± 0.80	2.9 ± 0.71
11. I have the opportunity to provide continuity of care	3.3 ± 0.66	3.0 ± 0.67
12. I have opportunities to acquire appropriate skills in practical procedures	3.2 ± 0.90	2.9 ± 0.76
13. My clinical teachers promote an atmosphere of mutual respect	3.0 ± 1.1	2.8 ± 0.86
14. I feel part of a team working here	3.0 ± 0.93	3.1 ± 0.71
15. I have protected educational time in this rotation	2.5 ± 1.0	2.3 ± 0.90
16. I have enough clinical learning opportunities for my needs	2.7 ± 0.96	2.8 ± 0.83
17. There is access to an educational program relevant to my needs	2.9 ± 0.87	2.7 ± 0.72
18. My clinical supervisor sets clear expectations	2.9 ± 0.98	2.6 ± 0.73
19. I have good clinical supervision at all times	2.7 ± 1.0	2.1 ± 0.94
20. I get regular feedback from seniors	2.6 ± 1.2	2.1 ± 1.2
21. Senior staff utilize learning opportunities effectively	2.5 ± 0.97	2.3 ± 1.0
22. My clinical teachers are well organized	2.8 ± 1.1	2.4 ± 0.92
23. The clinical teachers provide me with good feedback on my strengths and weaknesses	2.5 ± 1.1	2.0 ± 1.2
24. I am able to participate actively in educational events	3.2 ± 0.72	2.6 ± 0.62
25. My clinical teachers are enthusiastic	3.0 ± 0.86	2.7 ± 0.82
26. My clinical teachers are accessible	3.0 ± 1.0	2.6 ± 1.1
27. My clinical teachers have good communication skills	2.6 ± 0.98	2.4 ± 0.83
28. My clinical teachers have good teaching skills	3.1 ± 0.82	2.7 ± 0.77
29. My clinical teachers encourage me to be an independent learner	3.2 ± 0.79	2.9 ± 0.69
30. There are adequate catering facilities when I am on call	0.92 ± 1.0	1.3 ± 0.97
31. I feel physically safe within the hospital environment	2.3 ± 1.2	2.5 ± 1.1
32. There is a no-blame culture in this rotation	2.1 ± 1.2	2.0 ± 1.1
33. This hospital has good quality accommodation for residents, especially when on call	0.63 ± 0.96	0.68 ± 0.86
34. I get a lot of enjoyment out of my present job	2.0 ± 1.1	2.1 ± 0.96
35. I have suitable access to careers advice	2.4 ± 1.0	2.3 ± 0.90
36. There are good counselling opportunities for residents who experience difficulty regarding their training in this rotation	1.5 ± 1.2	1.4 ± 1.1
37. My clinical teachers have good mentoring skills	2.7 ± 0.89	2.3 ± 0.98
38. There is sex discrimination in this rotation	3.4 ± 0.69	3.3 ± 0.67
39. There is discrimination in this residency based on my ethnicity or religion	3.4 ± 0.85	3.1 ± 0.94
40. I have good collaboration with other residents	3.3 ± 0.75	2.9 ± 0.76
Total PHEEM	98.22 ± 38.09	
Role Autonomy	34.25 ± 13.69	
Teaching	39.7 ± 13.81	
Social Support	24.27 ± 10.59	

There were significant gender differences in the individual scores for six items in the perceptions of teaching and one item in the perception of social support domain (Table 3). Across all six items in the teaching domain, males consistently ranked their perceptions of teaching higher than the females. For the single gender difference in the domain for perception of social support, males rated higher scores for good collaboration with other residents (Table 3).

**Table 3:** Comparison of PHEEM items between male and female postgraduate trainees

*Characteristics*	*Males (Mean ± S.D.)*	*Females (Mean ± S.D.)*	*p-value*
*General items*			
Total PHEEM	101.02 ± 39.95	95.41 ± 36.23	.985
Role Autonomy	34.17 ± 14.76	34.33 ± 12.62	
Teaching	42.2 ± 14.35	37.2 ± 13.27	
Social Support	24.65 ± 10.84	23.88 ± 10.34	
*Specific items*			
**#19**: I have good clinical supervision at all times	2.7 ± 1.0	2.1 ± 0.94	**.005**
**#20**: I get regular feedback from seniors	2.6 ± 1.2	2.1 ± 1.2	**.037**
**#23**: The clinical teachers provide me with good feedback on my strengths and weaknesses	2.5 ± 1.1	2.0 ± 1.2	**.028**
**#24**: I am able to participate actively in educational events	3.2 ± 0.72	2.6 ± 0.62	**.002**
**#26**: My clinical teachers are accessible	3.0 ± 1.0	2.6 ± 1.1	**.049**
**#28**: My clinical teachers have good teaching skills	3.1 ± 0.82	2.7 ± 0.77	**.036**
#40: I have good collaboration with other residents	3.3 ± 0.75	2.9 ± 0.76	**.029**

In all the statistically significant comparisons shown, males consistently scored higher than females on the items shown.

When postgraduate trainees’ responses were compared by the sources of funding for their training, significant differences existed for one item in the domain for perception of teaching (item 27). Significant differences also existed by funding source for three items in the perception of social support (Table 4).

**Table 4:** Comparison of PHEEM items between funding source for postgraduate trainees’ medical education (Self-funding, Government scholarship, and Other)

Characteristics	Self-funding (Mean ± S.D.)	Government scholarship (Mean ± S.D.)	Other (Mean ± S.D.)	*p*-value
*General items*				
Total PHEEM	96.58 ± 38.88	100.18 ± 43.25	106.7 ± 35.22	.999
Role Autonomy	33.32 ± 14.06	32.74 ± 15.44	37.5 ± 13.44	
Teaching	39.8 ± 14.36	41.6 ± 16.62	42.7 ± 11.84	
Social Support	23.46 ± 10.46	25.84 ± 11.19	26.5 ± 9.94	
*Specific items*				
#27: My clinical teachers have good communication skills	2.4 ± 0.96	2.8 ± 1.1	2.8 ± 0.64	**.016**
#30: There are adequate catering facilities when I am on call	0.80 ± 0.83	1.4 ± 1.2	1.4 ± 1.1	.03* .06**
#33: This hospital has good quality accommodation for residents, especially when on call	0.46 ± 0.86	0.44 ± 0.63	1.3 ± 1.0	**.001**^**a**^ **.004**^**b**^
#35: I have suitable access to careers advice	2.2 ± 0.97	3.0 ± 0.63	2.5 ± 1.1	**.04**

*Significant comparison between Self-funding and Other funding

**Significant comparison between Self-funding and Government scholarship

^a^Significant comparison between Government scholarship and Other funding

^b^Significant comparison between Self-funding and Other funding

### Qualitative findings

Analyses of the transcribed data showed 5 major themes with a number of sub-themes as indicated in Table 5.

**Table 5:** Themes and sub-themes from the focus group discussions

Aspect of engagement	Sub-themes (“How do trainees’ perceptions contribute to engagement)
Trainee support	Support from peers and supervisors Regular interaction with supervisors Emotional support Mentorship Orientation during training
Supervision environment	Trainee autonomy Provision of feedback Support of trainee activities
Engagement with overall learning environment	Trainee-centered learning Availability of patients as a resource for learning Diverse perspectives from faculty Orientation on opportunities post-training Collegial faculty
Preparation for future practice	Learning environment provides suitable preparation Exposure to research prepares for future practice Desire additional learning will provide additional preparation Desire better resources to enhance training
Challenges that impede engagement	Challenges in training support structures Limitations in physical infrastructure Integration of work responsibilities and learning Limitations in training frameworks

### Trainee support

Many trainees aspire to certain ideals during and after training. Seeing their transition from novices to proficient practitioners of their specialties is exciting and motivates individuals along their career paths despite the uncertain journey from novice to proficient practitioner, perhaps even mastery. Participants acknowledged the importance of support, both emotional and intellectual, during training. One participant observed:

*“…we hold different activities within the department including case conferences and journal clubs and therefore these are done in presence of all seniors most of the time, and so they keep guiding us and teaching us…yeah, so that we improve at the next presentation.”*

Most of the participants valued the support given by peers and supervisors during training. One trainee in Pathology pointed out:

*“...[availability of supervisors] gives us a chance to always interact with them on a daily basis right from the time of lectures, to reading slides, the time of signing out cases.”*

One of the ways many trainees felt supported was through orientation when they started their training and during different periods of their training. Trainees valued the orientation that was provided on the educational goals, expectations, structure of the clinical services at their training stations, and opportunities available after training. One trainee observed,

*“…we get to learn our limits… we learn our limitations…as residents, we get to know at what point do we get to call the specialists, there are actually protocols where some of them are clearly put. A specialist needs to be called, informed about this case within this and this time.”*

### Supervision environment

For many residents, being able to work independently but with supervision from faculty provided an opportunity to satisfactorily engage with their supervisors during their training. Many residents appreciated the importance of the supervision of their teaching activities when they prepare to make presentations:

*“Seniors are there to see how you have prepared your slides, how you present your material and how you, ah, emotionally handle yourself.”*

Moreover, many residents highlighted the significance of their seniors and academic supervisors as role models in their academic preparation to practice.

### Engagement with overall learning environment

Many residents perceived that overall they were satisfactorily engaged with the learning environment and recognized that the environment provided insights into mistakes made during training. One trainee observed,

*“When I reflect back I see things that I, I could actually...if faced now I actually do differently, I face with confidence and I can tell what...I feel more in charge [pauses] personally as a postgraduate student.”*

Aspects of the learning environment that were perceived as enhancing training and practice included the availability of patients as a resource, collegial faculty, learning that was trainee-centered, and orientation on opportunities after they completed their training.

Many trainees perceived their learning environment as building their competencies:

*“…largely the hands-on experience we have in the department, it’s quite practical in the different aspects of the study, so that’s a good thing. The second is that most, majority of the aspects in studying, uh, are sort of student-led, and I think that is a good thing as opposed to just being fed information.”*

Trainees appreciated the collegial environment present in their clinical training. One participant commented,

*“…you will find a senior on the ward, and they are also very friendly to us, they treat us like we are colleagues, and also they mentor us, yeah. They have formed mentorship groups and our mentors look out for us so much.”*

### Preparation for future practice

Participants perceived the learning environment as one that provides a suitable preparation for their future practice despite some of the challenges such as heavy workloads. One participant observed,

*“I have really got a chance to see many cases which I am really happy about, so despite the heavy workloads, I think it uh, that prepares me for my next uh...phase.”*

Participants felt strongly that in spite of the limitations of training equipment and information resources, the availability of a rich patient resource prepared them for their future practice in their specialty. A participant from Internal Medicine commented:

*“I think anyone who has gone through this system will probably do well out there, because uh, you know, eh, patients is also a resource* (sic)*, and the condition they present to us is also a resource…”.*

Another participant from Surgery department:

*“…most of the conditions that we would wish to be exposed to they are readily available and we never lack the number of patients that we are interested in.”*

### Challenges that impede trainee engagement

Intertwined with participants’ aspirations and ideals were perceptions of challenges relating to engagement during training and preparation for future practice, which was a major theme.

The first subtheme included challenges related to training support structures. Some residents expressed dissatisfaction with interpersonal relationships between themselves and their academic faculty: One trainee commented,

*“…some lecturers lack proper communication skills, eh, so they push you into depression because you are stressed all the time, you can’t breathe, you are scared to enter the hospital because each day you are looking up to another day where you are going to be rebuked, and in a rather, um, immature way…yeah.”*

Another trainee observed, *“You are either stressed by the lecturer or by the nurses, because as a resident you know this is an emergency or not. You will see a patient and think, I will review them later. But then the nurse wants you to see them at that very minute and if you don’t see them they go and report to your lecturer who is going to grill you, yet that very lecturer wants work the next morning. So...it’s quite taxing and very depressing.”*

A few trainees perceived the inadequacy of orientation at the start and during their training as a challenge. One trainee observed,

*“…unfortunately orientation is just a two-hours’ talk and that is all. Afterwards it is baptism by fire and even for the students who don’t know how things go here it is usually very difficult.*

The second subtheme involved trainees’ perceptions that the limitations in physical infrastructure were a challenge in their training. These limitations included limited physical space, limitations in the availability of equipment during training, and inadequate exposure to resources routinely used in their specialties.

One participant observed,

*“I feel like if it was...all these resources were available to us more often we would get a richer experience and having, you know, that specialist training on them.”*

Another participant observed, *“…as a specialist in training, there are some, common, uh, investigations, that we should be able to familiarize ourselves with, I am thinking echocardiography, ECG, and these should be readily available to us, but unfortunately, they will guide me if I am wrong, we don’t get that.*

A third subtheme involved many trainees recognizing challenges with integrating work responsibilities and learning, and considered the balance unsatisfactory. One trainee remarked,

*“…you find we have a lot of clinical work, most colleagues who are on certain stations can be in clinical work all day, until late evening, 6.30[p.m.] when you are…if you are lucky to break off at that time, if you are not on call. But if you are on call, you will continue your duty until tomorrow, and tomorrow will still be the same similar day. You will continue your duty. So, if this person has a lecture, he’ll miss.*

The last subtheme involved challenges with limitations in training frameworks and policies. Although many trainees perceived that they were familiar with their limits during training, some decried the deficiency in communication and guidance during their training. One trainee commented,

*“…I don’t think our roles and even the resources were made clear...uh...as for me I learnt on job, many, many things, and generally in terms of general resource, ah, provision and preparation… we are not generally guided even before the course on where to go and find information… personally struggled to get access to information…”.*

Some of the trainees perceived the lack of clear policies regarding their training as a challenge that impeded their training. A trainee remarked,

*“…it seems like the ones who are supposed to inform of our limitations also don’t know how far the residents are supposed to go, eh. Ah, for example, like you can do something…um, one senior thinks it is okay, and then the other senior thinks it is not okay. So you get confused, eh, you are torn in between…am I okay, am I supposed to do this, eh?”*

## Discussion

The goal of this study was to explore the perceptions of postgraduate medical trainees towards their clinical learning environment in a resource-limited setting, and identify how these perceptions contribute to their engagement during training. Most resource-limited settings occur in developing low- and middle-income countries, and are characterized by insufficient infrastructure (or have rudimentary infrastructure), capacity building resources (or have limited capacity), financial, human, and other resources necessary to teach, supervise and mentor trainees [21]. In the context of this study, career engagement refers to the contextual capacity of the postgraduate trainees to navigate their career aspirations and successfully engage with their work through dynamic cognitive, emotional, and challenging interactions with their learning environment [22].

Our finding of an overall positive clinical learning environment but with room for improvement, showed better educational environment measures than some recent studies in Ethiopia among internal medicine residents, in Sudan among pediatric residents, and among multiple resident specialties in Nigeria and Morocco with similar economic status [12, 23–25]. Our study showed higher total and sub-domain scores than for these studies done in similar low-resource settings. We found a high internal reliability of the PHEEM tool. Our study was similar to a recent study by Shah and colleagues in Kenya among residents of 8 clinical specialties, whose overall perception of the clinical learning environment was more positive than negative [13]. Our total and sub-domain scores were, however, lower compared to studies done in high income countries including in Singapore and Ireland [26, 27].

The reason for gender differences in the responses to perceptions of teaching and social support in this study is unclear, given that males consistently ranked several items in the perception of teaching, and one item in the perception of social support higher than females. Considerations are needed to address the gender differences in the CLE during training, for example, providing targeted mentoring and support, and specific diversity, equity, and inclusivity (DEI) activities. To offset the gender differences seen in this study, one feasible solution could be through establishing a DEI committee that is contextually appropriate, wide-ranging, and relevant to the education needs and experiences of trainees. A blueprint for such a DEI committee can be adapted from recent studies [28–30]. It is remarkable, also, that both male and female postgraduates in this study endorsed a high perception of the absence of discrimination based on sex, ethnicity or religion. In a recent cross-sectional study among medical students at a South African university, discrimination was common and pervasive, with almost two-thirds of study participants reporting gender and racial discrimination, especially among female trainees [31]. The result from this South African university study contrasts with our findings. Certainly, even though the clinical learning environments might be similar in many ways, there are also differences in the physical environments between the South African (upper middle-income) study and the Ugandan (lower income) one, these being divergent in their World Bank country classifications [32]. Nevertheless, the perception of discrimination among postgraduate medical trainees in this study differed from that of trainees in higher income settings in Africa and the Western world.

We included funding source for the postgraduate trainees’ education, a characteristic not routinely described in literature, and perhaps is described here for the first time in relation to the clinical learning environment. Funding potentially impacts trainees’ welfare during daily living and through the training duration. Postgraduate trainees who fund their own training consistently rated items pertaining to catering and accommodation while on call, and access to career advice, lower than those who had scholarships from the government or other external funding.

Qualitative data in this study complemented the quantitative data from the PHEEM survey. Our qualitative findings differed from a study done among Internal Medicine residents at a medical college in Addis Ababa in Ethiopia where trainees perceived the clinical learning environment as mostly negative [12]. Fisseha and colleagues reported that academic faculty were perceived as being non-supportive towards trainees’ learning, providing inadequate bedside teaching, little contact with trainees during ward rounds, and giving inadequate feedback [12]. Conversely, our results showed that residents felt supported by their supervisors during training, and appreciated the autonomy given to them while being supervised in their learning activities.

Residents in the study by Fisseha and colleagues perceived the resource limitations and inadequate diagnostic and therapeutic interventions as significant impediments to their training and preparation for future practice and expressed feeling unready to practice as consultants on completing their training [12]. In our study, however, despite training in a similar low-resource setting, participants perceived their training as providing a good preparation for future practice; the availability and exposure to a rich patient resource was perceived as a suitable preparation despite some of the limitations of equipment and other resources. Participants also perceived that overall their interactions with the clinical learning environment were satisfactory.

Consistent with another study in which residents perceived that their supervisors engaged them individually and provided multiple perspectives and feedback, participants in our study appreciated the regular interaction with supervisors, emotional support, and mentorship during their training [14]. Similar sets of challenges including work-life balance, and insufficient resources such as equipment, space, and supervisors, were encountered in this study and in the study by Erumeda and colleagues [14].

Many of the themes arising from a recent workshop on Family Medicine training in Africa, especially interactions between trainees and supervisors and feedback, were shared by trainees in our study [33]. In particular, regular interactions between trainees and their supervisors, and participation in educational activities under supervision were perceived as enhancing the learning of trainees. These educational activities included case presentations, journal clubs, and practical procedures supervised by academic faculty. In our study, peer interaction was another strong point of the learning environment. Trainees acknowledged the regular presence and support of senior residents who were available to consult, and provided guidance and counsel during calls, ward rounds, and in the clinics.

Perceptions regarding availability of resources for training were similar to that in a recent study from South Africa in which family medicine registrars (residents) reported shortages of resources such as training tools (e.g. ECG), Internet availability, and training space [34]. In both studies, residents observed that essential equipment were unavailable and hindered their training because they perceived that they could not develop mastery of skills needed in working with the lacking equipment.

## Study strengths and limitations

This study contributes data on the international utility of the PHEEM in evaluating the clinical learning environment in low-resource settings and the qualitative findings triangulated the PHEEM data. One of the major strengths of our study was that it had postgraduate trainees from multiple specialties, which contributed a greater depth to understanding the clinical learning environment through both the PHEEM survey and the qualitative study. Another strength was the use of both quantitative and qualitative approaches, with the latter complementing the findings from the survey. Our study findings, however, are limited by its cross-sectional design, and the outcomes may not generalize to another time or place; the findings, nonetheless, provide baseline information that will be useful for future studies of this nature. Another limitation is the absence of systematic sampling. It is possible that some of those who decided to participate in the survey did so because of an unanticipated bias. Lastly, some departments had small numbers of one gender, and the perceived differences in responses may not be wholly representative of the clinical learning environment in those departments.

## Conclusion

Regardless, the conclusion from these findings is that the clinical learning environment is positive despite limited infrastructure, human, and other resources. Trainees’ insights provided data on several potential areas that could be modified or endorsed to improve the learning environment in our study context. Key elements of the CLE that will need improvement include training support, physical infrastructure, training frameworks, and supervision. Further studies are warranted on these elements and on the gender differences in the perceptions of aspects of the CLE that would enhance training.

## Data Availability

The datasets used and/or analysed during the current study are available from the corresponding author on reasonable request.

## Abbreviations

CLE: Clinical learning environment
DEI: Diversity, equity, and inclusivity
FGD: Focus group discussion
MUST: Mbarara University of Science and Technology
PHEEM: Postgraduate hospital education environment measure
